# A Novel Algorithm for Independent Component Analysis with Reference and Methods for Its Applications

**DOI:** 10.1371/journal.pone.0093984

**Published:** 2014-05-14

**Authors:** Jian-Xun Mi

**Affiliations:** 1 Chongqing Key Laboratory of Computational Intelligence, Chongqing University of Posts and Telecommunications, Chongqing, China; 2 College of Computer Science and Technology, Chongqing University of Posts and Telecommunications, Chongqing, China; University of Ulm, Germany

## Abstract

This paper presents a stable and fast algorithm for independent component analysis with reference (ICA-R). This is a technique for incorporating available reference signals into the ICA contrast function so as to form an augmented Lagrangian function under the framework of constrained ICA (cICA). The previous ICA-R algorithm was constructed by solving the optimization problem via a Newton-like learning style. Unfortunately, the slow convergence and potential misconvergence limit the capability of ICA-R. This paper first investigates and probes the flaws of the previous algorithm and then introduces a new stable algorithm with a faster convergence speed. There are two other highlights in this paper: first, new approaches, including the reference deflation technique and a direct way of obtaining references, are introduced to facilitate the application of ICA-R; second, a new method is proposed that the new ICA-R is used to recover the complete underlying sources with new advantages compared with other classical ICA methods. Finally, the experiments on both synthetic and real-world data verify the better performance of the new algorithm over both previous ICA-R and other well-known methods.

## Introduction

Independent component analysis (ICA) is a data analysis technique for uncovering independent components (ICs) which underlie the observational data [1] [2] [43]. This technique finds a mutually independent representation of the original data by seeking a linear transformation. Let us denote an *n*–dimensional observed signal by 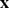  =  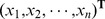, which is a linear mixture of the independent components, say 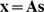, where 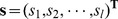 (usually 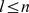) and 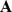 is an 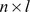 linear mixture matrix. And let 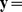 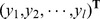 denote an *l*–dimensional signal, which is the estimated vector of independent components (ICs). Generally, the ICA problem can be expressed as the following linear relationship: 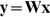
(1)where 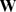 is an unknown 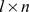 matrix of full rank, which is the demixing matrix for which ICA is seeking. For the sake of convenience, here 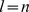 is assumed first.

Most ICA algorithms attempt to recover the same number of ICs as the observed mixture signals, i.e. complete ICA. However, in several practical ICA applications only portions of underlying ICs are interesting while conventional ICA algorithms have to compute all sources, which is an exhaustive extraction process and time-consuming sometimes. In many cases, some prior information is available to distinguish ICs of interest, especially for biomedical signal processing problems. For example, in the case of applying conventional ICA to remove artifacts in electroencephalogram (EEG) or magnetoencephalogram (MEG), the necessary manual selection of ICs corresponding to artifacts may be inconvenient and unreliable. A practical alternative is to incorporate a priori knowledge as an additional constraint into classical ICA algorithms. Generally the constraints may be adopted to reduce the dimensionality of the output of the ICA, hence only the desired ICs are isolated.

Recently a new technique referred to as ICA with reference (ICA-R) has been proposed under the constrained ICA (cICA) framework [3]–[5] [40] [41] [42], to separate a desired subset of ICs by utilizing prior information as the reference signal. Some earlier studies [6] [7] [8] also have used reference signals to extract the sources. The framework of cICA forms an augmented Lagrangian function consisting of the ICA contrast function, equality constraint, and inequality constraint, where the last two are with regard to additional requests and the incorporation of prior information, respectively. The Newton-like learning approach is proposed to give an optimal solution to this optimization problem. So far, the ICA-R algorithm has been used in different applications [8]–[17] [40] [41], which justifies the validity of this technique. The prior information such as interesting frequency, waveform, and so on, can be used to create reference signals so as to constrain the output to be the required ICs. For example, an approach was proposed by Zhang [18] to construct a reference signal for weak temporally correlated source. A fast algorithm for one-unit ICA-R was proposed by Lin et al. [19] in which normalization of the weight vector at each step is used to expedite the convergence process, instead of using the equality constraint.

In [3] and [5] by Lu and Rajapake, the local convergence stability of ICA-R is verified by showing that the optimum output from their ICA-R algorithm can satisfy Karush–Kuhn–Tucker (KKT) conditions locally. However the existing ICA-R algorithms could not ensure the global convergence, because under the cICA framework the convexity of the Lagrange function is not guaranteed.

Firstly, this paper is to reveal the flaws of the previous ICA-R algorithms, that is, they may converge to a fake point and output artifact IC, which is referred to as misconvergence. And then we present a new algorithm which ensures that the output of ICA-R is a true required independent source. Secondly, this paper proposes several approaches for simple and convenient applications of ICA-R, including a way of directly obtaining references from observed channels and reference deflation techniques for easy parameter selection. Thirdly, this paper aims to extend new ICA-R to extract entire underlying components, which has superiority over FastICA in maintaining structure of the original sources and is with the capability to assist in identifying the number of underlying sources.

The paper is organized as follows. Section II reviews classic ICA algorithms, the ICA-R algorithm and its fast algorithm briefly at first, then investigates and analyzes the cause of the misconvergence of previous ICA-R methods. In Section III, a new algorithm for ICA-R is proposed. Section IV discusses the approaches which could facilitate the application of the new ICA-R algorithm and extend the application to recover all underlying ICs. In Section V, the experiments using both synthetic data and real-world signals demonstrate the performance and superiority of the new algorithm. Section VI concludes the paper with some discursive remarks.

## Previous algorithms

### Classic ICA algorithms

Several algorithms were developed to realize ICA. Pham et al. [20] formulated the likelihood for noise-free ICA which was estimated by a maximum likelihood method. In [21] [22], algorithms were derived from a neural network viewpoint and based on maximizing the output entropy with non-linear outputs. Nonlinear principal component analysis was used to perform blind source separation (BSS) in [23] and the recovered sources of which is believed to be ICs. The JADE algorithm [24] was presented to solve the ICA contrast function in terms of a sum of squared fourth-order cross-cumulants. Based on the concept that non-Gaussian is independent [25], ICA also can be performed by maximizing the negentropy which is a measure of non-Gaussianity. Approximations of negentropy were presented in [26] which avoid the difficulty of estimation of negentropy and the non-robustness of the kurtosis. FastICA [27] [28] is the most popular ICA algorithm which maximizes the negentropy to recover all ICs with low computational load and application convenience [29]. Since our proposed algorithm [4] [5] also adopted negentropy as contrast function, we pay more attention on comparing the new algorithm with FastICA than others.

FastICA optimizes the contrast function via a fixed-point learning method which makes the convergence very rapid. For extracting several ICs, it is necessary to prevent the different vectors from converging to the same maximum. Hence, two available techniques, the deflationary orthogonalization approach and the symmetric orthogonalization approach, are used by FastICA. However for the deflationary orthogonalization approach, which recovers ICs one-by-one the earlier recovered ICs are “privileged” over the later. The estimation errors existed in the recovered vectors are accumulated in the subsequent ones. Alternatively, the symmetric orthogonalization process can be used to estimate ICs in a parallel manner. But the explicit decorrelation process is usually too strong that it slows the convergence of algorithm. Furthermore, these two approaches force the outputs of FastICA to be uncorrelated which could distort original data if some empirical underlying sources are not perfectly independent.

Before implementing FastICA, principal component analysis (PCA) is used to pre-whiten the data and remove the components with very small eigenvalues, thought to be noise. To avoid losing real ICs in the mixture, the principal components are used to save as many as possible, however this may cause the inclusion of the noise components. Since FastICA has to estimate the same number of the outputs as the number of the inputs produced by PCA, the result will contain noise components. Moreover, the estimation of the noise components is not only time-consuming but can also harm the true ICs by orthogonalization.

### Previous ICA-R algorithms

Considering that there is a priori knowledge about the desired ICs, ICA-R utilizes such knowledge by incorporating the prior information in the form of additional equality and inequality constraints into the ICA contrast function, as given in [12]. Then ICA-R aims to produce the desired subset of ICs without the post-selection which is needed in classical ICA methods. According to the studies by Lu and Rajapake, the mathematical account of ICA-R algorithm is presented in detail in Appendix 1.

In empirical applications, one-unit ICA-R, extracting one IC at a time with the corresponding reference signal, is preferred for the sake of simplicity and straightforwardness [8] [10]. When several ICs are expected, this can be done by repeated runs of the one-unit ICA-R algorithm. Hence in the following parts of this paper only a one-unit ICA-R algorithm will be considered. In our previous work [11], the one-unit ICA-R algorithm was improved in rigidity; at the same time, some principles were given for proper selection of closeness measurement functions. By employing correlation 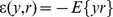 and mean squared error (MSE) 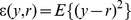 as the closeness measurement (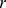 is of unit variance), two learning rules suggested in [11] are given respectively by: 
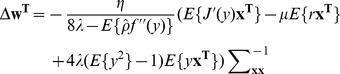
(2)and 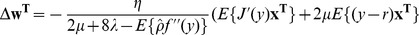
(3)where 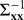 denotes the inverted covariance matrix of the observed signal 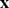 (If 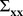 is near-singular, it can be transformed into a non-singular matrix by a whitening process, e.g., PCA); 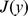 is the negentropy of a signal 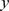; 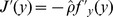; 
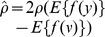
; 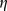 is the learning rate which is fixed; 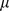 and 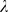 are Lagrange multipliers with the respective gradient ascent learning rules as: 
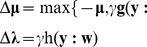
(4)where 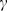 is the learning rate.

A fast one-unit ICA-R algorithm was proposed by Lin et al., in [19]. Compared with the original one-unit ICA-R algorithm, one aspect of the improvement for the fast one-unit ICA-R algorithm is to make the observed signals pre-whitened so as to let the covariance matrix equal the identity matrix, which can expedite the convergence. Other aspect for the improvement is that the equality constraint is replaced by normalizing the weight vector 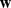. The corresponding update rules for 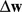 is similar to Eqns. (2) and (3), nevertheless, where 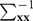 is an identity matrix and the equality constraint is absent. From the experimental results given in [19], it can be seen that compared to the original ICA-R algorithm, the fast algorithm spends approximately half of its time extracting the desired ICs of the same quality.

### Analyses of the convergence stability of ICA-R algorithm

The investigation of the global convergence of previous one-unit ICA-R algorithm is presented here. First, we make some statements on parameter choice. As a closeness measurement, the philosophy for the inequality constraint being incorporated is to constrain the ICA contrast function so as to make it converge to the desired IC. Therefore, only the IC which is in the neighborhood of a given reference signal defined by 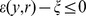, where 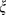 is a threshold and 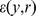 measures the distance, can be extracted by ICA-R algorithm. The threshold 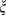 is the most important parameter for the ICA-R problem. Generally, the right value of 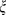 should be lain in the following range: 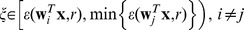
(5)where 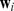 is the required demixing vector and 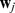 is the vector corresponding to the any other ICs. If 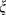 is below the range, then none of ICs can satisfy the constraint, i.e. there is no IC within the feasible range defined by the inequality constraint (the possible output is not a real IC); and if 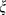 is selected beyond the upper bound of the range, then the output 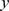 could be the undesired IC because more than one local minimum is within the inequality feasible range. A practicable way for selecting 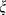 was given in [3]. In the rest of this subsection, the eligible threshold parameter 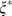 satisfying Eqn. (5) is considered to analyze the stability of ICA-R algorithm. Meanwhile, 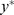 is denoted as the optimum output of interest.

**Figure 1:**
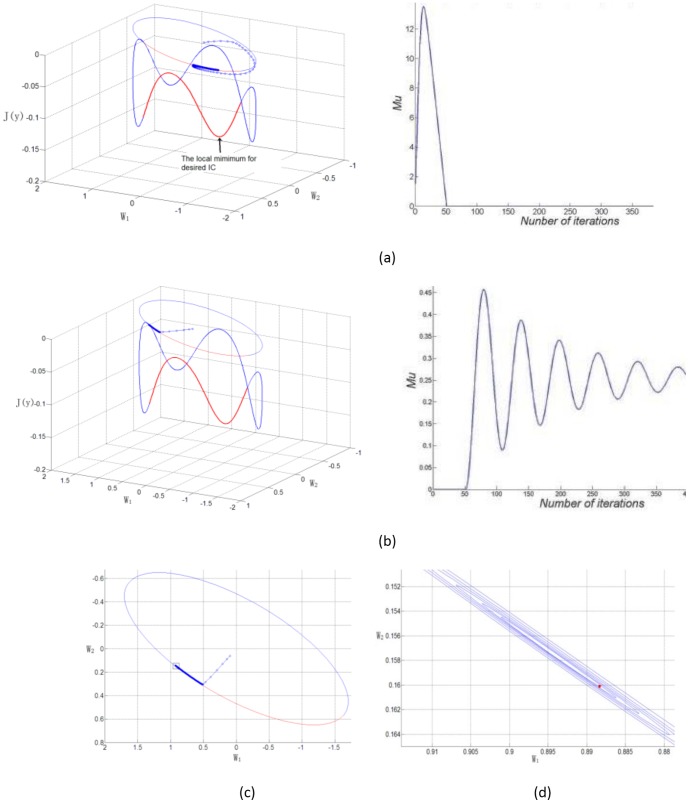
Illustration of two typical learning traces of demixing vector by previous ICA-R algorithm. (a) An example of accurate convergence for desired demixing vector (left) along with the evolutions of 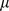 vs the number of iterations (right). On 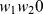 plane, each step of 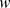 is presented by small cycles and linked by a line; and the ellipse is the confine defined by equality constraint. Below the 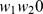 plane, the curve stands for the values of 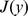 for projections as a function of ellipse. The red line highlights the region fulfilling the inequality constraint. (b) An example of misconvergence (left) where the algorithm is trapped around the inequality constraint border along with the corresponding evolutions of 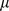 (right). (c). 2-D illustration of the misconvergence example on 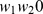 plane. (d) The magnification of the black box in (c). The image of magnification manifests that the learning trace librates and stops at the red dot (the blue cycles are removed for visual convenience).

In this paragraph we analyze the global convergence of the algorithms given in Eqns. (2) and (3). Particularly, the point that should be stressed is that the following analyses are valid for the algorithms with other closeness measurements as well. First we review the principle and conclusion of stability analysis in previous ICA-R literatures. According to Lu's analyses, the optimum triple 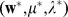 for KKT conditions [30] [31] is defined which satisfies the first order conditions: 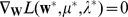, 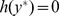, 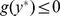, 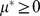, 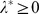, where 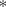 indicates the optimum value; and the stability at the global optima is examined by testing the positive-definiteness of the Hessian matrix, which is approximated by 
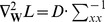
 in ICA-R algorithms. The Hessian matrixes (approximate Hessian matrixes exactly) of the algorithms with Eqns.(2) and (3) are 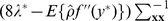 and 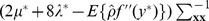; and they are always positive-definite because the input covariance matrix 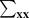 is nonsingular (if not, PCA can be used to help it become nonsingular) and the elements of 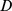 are greater than zero (for 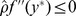, and all the Lagrangian multipliers are greater than zero). The local stability of minimum of the ICA-R algorithm therefore is confirmed. However, can we affirm that the Newton-like learning rule will reach the optimal output 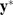 by proving the local satisfaction of the KKT Condition? In Figure 1, we illustrate typical examples of learning processes for the ICA-R algorithm with Eqn. (2), where it can be seen that a counterexample is against Lu's standpoint.

Let us present the details of examples in Figure 1. Assuming that two normalized synthetic ICs, 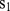 and 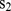 are used, with the kurtosis values of −1.2910 and −1.4982, respectively (where 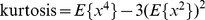), and are mixed with the observed data by a matrix 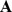:
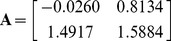



Thereafter, a normalized reference signal is created whose closeness values, measured by 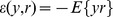, between each of the two ICs are −0.8907 and −0.4166, respectively (the normalization of a random variable means to remove its mean and let it have unit variance). The threshold 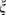 is set to −0.6 so that only 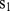, the supposed desired one, can satisfy the inequality constraint that 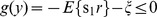. Here, we denote the weight vector as 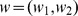. With a small random initial weight vector, however, the previous ICA-R algorithm has two different typical learning traces. Figure 1 (a) gives an example of successfully discovering the accurate demixing vector where the final vector is at the point around 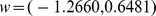 on which the observed data can project to obtain the desired IC; meanwhile, a counterexample, called misconvergence, is shown in Figure 1.(b), where the algorithm fails to find the minimum and is trapped around the inequality constraint border. As shown in Figure 1, the damping is a faulty behavior and oscillations also can be persistent in other cases. The cause whether oscillations are persistent or damped mainly relates to the position of the feasible boundary. However, the actual situation is more complicated when considering the tripartite actions by contrast function and two types of constraints. Further discussion of this is beyond the scope of this study.

To investigate the cause of misconvergence, the two graphs in Figure 1(c) show the obviously different evolutions of Lagrange multiplier for inequality constraint, 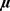. The left graph (in the case of a successful extraction), where after a rise 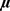 decreases to 0, indicates that the learning process is led into the feasible area by inequality constraint exactly; but the oscillation shown in the right graph (for the case of failure learning) indicates that the learning process steps into and out the feasible region repeatedly (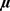 increases when the weight vector is out of the feasible region and decreases when in feasible region). Furthermore, the right part of Figure 1(d) shows the details of last steps of the failure learning process, which confirms that the algorithm vibrates at the wrong point around (0.8874, 0.1583).

What leads to the failure? Commonly utilizing Newton-like learning rule to solve the augmented Lagrangian function of ICA-R, the inequality constraint is considered to draw the learning into its own feasible region by increment of 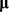 as the weight vector is out of the feasible region. When stepping into the feasible region, the 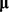 will descend to 0, i.e. the inequality constraint will be suspended. In particular, there is another similar situation that the initial weight vector is casually in feasible region so that the inequality constraint would not be activated at all. As 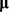 decreases to a small value, the algorithm will search towards the direction for decreasing the value of 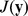 in the feasible space of equality constraint. Unfortunately, it is possible that the weight vector is out of the basin of attraction of the desired IC when the inequality constraint has been inactivated already. Under this circumstance, the learning will head towards the other ICs; then stepping out of the feasible region once, the inequality constraint will be activated (i.e., 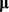 is increased); subsequently the learning turns back into the feasible region again (i.e., 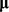 is decreased). However, it still could not be in the attractive region of the desired IC so that the oscillation starts.

The simple counterexample above indicates that the local stability analysis is not sufficient. By mathematical analysis and experimental investigation, it was found that the ICA-R algorithm can find the desired optimum point only if the algorithm steps into its corresponding attractive basin. But under the cICA framework, it cannot ensure that the feasible region of inequality constraint is within the basin of attraction of desired IC. In other words, the inequality constraint cannot definitely force the learning process to step into the attractive region of the desired IC. Given the valid parameters which let the desired IC be the only local minimum in feasible region, the probability that the ICA-R algorithm misconverges is approximately in proportion to the ratio of the part of inequality constraint beyond the scope of attractive basin to the part in the scope of attractive basin. The attractive basin of the desired IC is related to the relative value of the ICA contrast function. In Appendix 2, the mathematical analyses reveal which IC the ICA-R algorithm converges to from a vector in feasible space of equality constraint, i.e. the scope of an IC's attractive basin. From the mathematical analyses, we can say that the smaller 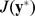 or the greater the negentropy is, the wider the attractive basin is. Hence, the IC with relatively larger negentropy is more likely to find the demixing vector generally. Meanwhile, the reference signal itself and threshold 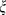 are the two factors for determining the center (please refer to Appendix 3) and the size of the feasible region of inequality constraint, respectively. To extract IC with small negentropy without misconvergence, we therefore do not only need a more reliable reference signal, which means that the reference signal should strongly correlate with the desired IC and be uncorrelated with the others, but also need a smaller feasible region of inequality constraint, which could be achieved by adjusting the threshold 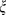. Nevertheless, the feasible region is generally difficult to be settled in advance.

More seriously, when extracting an IC from a mixture consisting of independent components of both super-Gaussian and sub-Gaussian distribution, it is possible that the algorithm will diverge for the negative definite Hessian matrix once the learning process is in the basin of other ICs with different distribution types. The cause should be that the term, 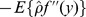, in the Hessian matrix which can be negative when 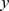 is far from 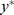.

Thus it can be concluded that ICA-R algorithm is not with the global capability of extracting desired ICs stably, though using legal parameters, but with probability of misconvergence. In other words, although the framework of the constrained ICA is known as a method to incorporate extra constraints into ICA contrast function, the Newton-like learning technique is not capable of finding the minimum determinately. Mathematically, it could be found that the sufficient KKT condition cannot be fulfilled in cICA framework, since it demands that the contrast function 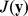 is convex in the feasible region of constraints which the cICA cannot guarantee which is illustrated by Figure 1. Furthermore, although replacing the equality constraint by normalizing the weight vector, there is no fundamental alteration for Lin's improved version of fast one-unit ICA-R algorithm which will still suffer from the defect of ICA-R.

In practical applications, it is risky to use the outcome of previous ICA-R algorithm in a run for the pseudo IC by possible misconvergence, especially in the case of the pseudo IC being quite similar to a true IC. It is suggested that the true IC should appear most in several runs of ICA-R algorithm [4]. Unfortunately, the understanding that the number of the outputs of real ICs is greater than the number of the outputs of pseudo ones is incorrect, which was supported by our experiments. Therefore, even doing the time-consuming reruns, the application of previous ICA-R algorithms would still confront the additional inconvenient point of discriminating the true ICs from the fake products.

## Methods

In this section, a new stable fast algorithm for one-unit ICA-R is presented. This algorithm is based on the consideration that there should be an added mechanism for the algorithm which is able to detect the possible failure of convergence at an early stage and to restart the algorithm with a more appropriate initial point. A simple and efficient method to detect future misconvergence is to examine the secondary increment of 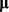 or the negative definiteness of the Hessian matrix (when 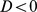). When ICA-R extracts IC successfully, the evolution of 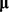 should be no more than one fluctuation, because the secondary increment of 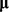 only means that the learning process steps out of the feasible region. Thus, once the secondary increment of 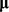 is detected, the algorithm should be restarted. At the same time, when 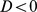, the algorithm should be restarted as well for the desired minimum cannot be found by negative definite Hessian matrix. Next, we introduce the deflationary orthogonalization technique to avoid the case that the next random initial 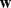 is with the same direction as the former ones. Once the algorithm is restarted, the new initial 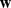 will be created to have an angle of round 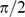 to all the previous used vectors via the Gram-Schmidt method [30], which could be expressed as: 
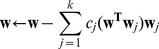
(6)where 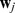 is one of 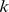 vectors which have already been used as the initial vectors but without extracting the desired IC.

Consequently, the stable fast algorithm of one-unit ICA-R can be summarized in Table 1. Based on the fact that Lin's fast one-unit algorithm converges more rapidly than Lu's, in our algorithm we also normalize the length of the weight vector in each iteration instead of using the equality constraint.

**Table 1:** The new fast one-unit ICA-R algorithm.

*Step* 1. Center the observed signals 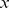 to remove its mean;
*Step* 2. Whiten the observed signals;
*Step* 3. Choose an initial value for 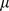 , generally let 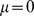 ;
*Step* 4. Take a random initial vector 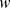 of norm 1;
*Step* 5. Update the Lagrange multiplier 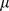 ;
*Step* 6. Update the demixing vector 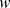 : 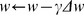 , where 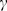 is the learning rate;
*Step* 7. Normalize the 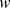 by 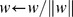 ;
*Step* 8. While detecting the secondary increment of 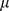 or a minus 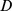 , then restart the algorithm from *Step* 3 with a new initial 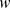 by deflationary orthogonalization technique;

**Remark 1**: If pre-whitening is not expected, *Step* 2 should be omitted and the normalization of weight vector will be replaced by 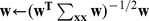 in *Step* 7.

## The Discussions on Applications of the proposed ICA-R algorithm

Methodologies introduced in this Section are to facilitate the application of ICA-R algorithm. The first subsection presented two methods to select parameter of the closeness threshold. Furthermore, instead of concocting a possibly complicated reference how to use one of the channels as the reference (helpful in some cases) is introduced in second subsection. In the third subsection, our new fast one-unit ICA-R algorithm is extended to estimate all the underlying ICs, which performs better than FastICA method. Since there is no essential difference between 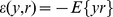 and 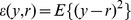 when 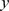 and 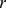 are with unit variance, the algorithm discussed in this section is assumed to use 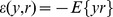 (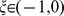) straightforwardly.

### Selection of 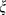


Usually the determination of the threshold parameter 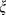 is most critical for ICA-R algorithm, since the closeness between the desired IC and the reference cannot be known in advance. There are two types of errors for missing selection of 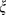: Firstly if 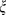 is selected to make the feasible region of the inequality constraint so small that none of ICs is within it, ICA-R algorithm will not converge to any IC. Secondly, if 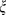 is selected to make the feasible region so large that other local minima are included, the ICA-R algorithm probably produces undesired ICs. It is more difficult, to some degree, for previous ICA-R algorithm to select a proper 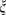 and determine the true IC, since even with right 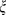 the previous algorithms may produce the fake ICs.

In this paper, we introduce two approaches to loose the selection of 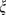. In this paragraph, we demonstrate the first one. To ensure true IC is extracted by our ICA-R algorithm, we should set 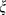 to be an appropriate big value; as thus the ICA-R algorithm may extract the ICs of no interest. Our remedy is if an undesired IC, say 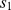, is produced, we can decorrelate it with the reference by: 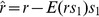
(7)


With the newly reconstructed reference 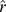, the next run of ICA-R algorithm will never converge to the first IC. The reason is based on the independent property that 
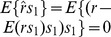
. We suggest that in the following run of ICA-R algorithm with new reference, 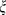, can be decreased a little in order to let the feasible region be smaller since the proportion of the desired IC in new reference increases. Under some worse circumstances, the other undesired ICs could be produced again. Thus, the approach in Eqn.(7) could be applied repeatedly to remove the undesired IC from the reference until the expected IC is obtained. However, considering some real world applications, the outcomes of maximum negentropy may be not totally independent, thus directly operating Eqn.(7) to remove the composition of the next IC from reference could cause the new reference to become correlated with those former removed ICs. For the case that more than one undesired IC (supposing 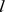 undesired ICs to have already extracted) needs to be removed from the reference, we should reconstruct the current IC to make it uncorrelated with the former extracted ICs in the first place by: 
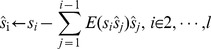
(8)where 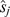 is the previously reconstructed undesired IC before being removed from the reference. This process can be replaced by PCA after each extraction; nevertheless it may take longer. Usually, even with reference of low quality or a rougher reference, less than three runs of ICA-R will produce the desired IC. For the above described approach, actually a deflation scheme [32], we name it as reference deflation method.

In other case when user cannot distinguish the expected IC and only want the IC which is “closest” to reference, we could assign a smaller value to 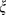 first so as to make the feasible region of inequality constraint smaller at first to assure no undesired minimum lies in the feasible region [3]. However, this will take the risk that the desired IC is out of feasible region as well. Fortunately, our algorithm can indicate this situation, since in such case our algorithm will not converge any more (the previous algorithm will probably produce fake IC). And to solve this problem, we can gradually increase the feasible region by decreasing the value of 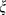. Once the algorithm converges, the produced IC must be the desired IC. We should remark that the above approach introduced in this paragraph would be time-consuming to discover that the algorithm is not convergent by examining whether or not the iterations of the algorithm reach a presetting large number.

### Providing ICA-R with a direct reference

Since reference signal is required for ICA-R algorithm to straightforwardly recover the desired IC, several methods have been developed to provide ICA-R algorithm with a suitable reference [8] [18]. In some cases, the frequency of the signal is the most important characteristic, so researchers invented some approaches to create periodical signals [8], [33] as reference. For example, sine waves can be used as reference. However, when constructing the periodical reference signal, there are at least three aspects which have to be carefully considered: (1) the frequency which is application-dependent; (2) the initial phase which needs a cautious determination; (3). the morphology of the reference where the empirical experience is also important. Even though the aforementioned three points are satisfied, in some situations the method that provides ICA-R with a periodical reference to extract a desired IC may still fail. The reason is that some underlying independent sources coming from complex system seem like periodic signals but actually are chaotic, which may be “far” from a periodic signal. On the other hand, it could be tough to concoct a proper reference to extract the aperiodic independent source. The designing of a feasible reference sometimes could obstruct the application of ICA-R so that some researchers would rather recover all ICs and then pick up their interested sources.

In many practical applications, it can be found that the desired ICs dominate some observed signal channels. In such cases, a simple and convenient way to perform ICA-R is to employ observed signal as reference directly. For example, to remove eye blink artifacts from the EEG channels, the reference could be one channel of the observed EEG which is badly contaminated by eye blink.

Let us denote a normalized signal channel by: 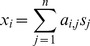
(9)where 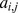 is the mixing coefficient that 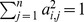 and we can suppose 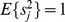. When the observed channel 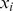 is utilized as the reference and 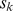 (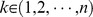) is assumed as the desired IC which is the major component of 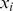, ICA-R can extract 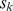 from the mixture with a parameter 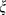 in the range of 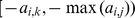, where 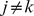. Since 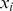 mostly consists of 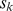, the mixing coefficient 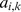 should be much bigger than the secondary biggest mixing coefficient, thus there could be a wide value range selection for the threshold parameter. If a reference channel consists of more than one major IC, the reference deflation technique can be used to help at extracting the desired IC.

### Recovering complete ICs by ICA-R

Complete ICA can be achieved by method of maximization of nongaussianity. At present, the most popular ICA method of maximization of nongaussianity is FastICA [28], which will be the counterpart to be compared with some characteristics of our method in this paper. When the ICs are independent, the mixing vectors produced by FastICA are orthogonal to each other. Hence, by the compulsive orthogonalization process, FastICA will not converge to the same ICs but recover all the ICs. The drawbacks of the compulsive orthogonalization can be found in Section II. A.

The new one-unit ICA-R algorithm proposed in this paper is capable of recovering all ICs one by one without error cumulation and compulsive orthogonalized outputs. Straightforwardly, let us assume that there is an observed signal channel consisting of all ICs which is served as initial reference in this case. Then, the threshold 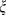 should be set to a big value, for example 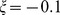, so as to assure that several ICs are in the constrained feasible region of inequality. The algorithm constructed for recovering all ICs is summarized in Table 2.

**Table 2:** The algorithm for recovering all ICs.

Step 1. Choose a proper 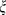 and an initial reference;
Step 2. Run ICA-R to extract an IC;
Step 3. Reconstruct the reference by reference deflation technique;
Step 4. If not all ICs are recovered, go back to Step 2.

Here, some commentaries are given for supporting our approach. After each run of one-unit ICA-R algorithm, a new reference is created by removing the new extracted IC from the former reference. This operation will change the feasible region of the inequality constraint so that the new region will exclude the extracted ICs and include some ICs which are out of the feasible region. Though ICs are recovered one by one in our algorithm, the subsequent ICs would not suffer from the errors of the first estimated ICs because errors only interfere with the reference. Moreover, by avoiding using compulsive decorrelation process to prevent the identical convergence, our algorithm prevents extracting the same ICs by deflating reference instead. Under certain circumstance where observed signals consist of only part of the whole ICs, the initial reference could be produced by summing several observed signals together which makes the reference more likely to contain all underlying sources. It is almost impossible but could occur that the ICs are counteracted or severely weakened in summation of channels. To avoid such possible neglect, we can construct different linear combinations of all channels and then remove the obtained ICs from them; the neglected ICs will emerge. On the other hand, the above approach is capable of revealing the composition of a concerned observed signal without extraction of all ICs when the signal is mixed by only a few ICs.

Attractively, this algorithm for recovering the whole ICs can still perform well when Gaussian sensor noise is present. In certain practical applications of ICA, the observed signal channels is more than the independent sources; meanwhile, the independent Gaussian observed noises are present in each sensor [34] [35]. Usually, PCA is expected to reduce the dimension of noise, but on the other hand taking the risk of abandoning the weak ICs. Hence, the principal components have to be reserved as much as possible. Then, the FastICA algorithm recovers all the remainder components as “ICs”, which not only makes the algorithm spend more time for estimating the noisy “ICs” and needs the post-identification to choose the real ICs but also abides the noisy ICs to harm the real ICs by orthogonalization [36]. However, theoretically, our ICA-R will not converge to the noisy sources after all real ICs are recovered (assuming that none of real ICs is of Gaussian distribution), because the negentropy value of Gaussian noise is equal to 0. Empirically, the negentropy of the noise components are not perfectly equal to 0, but still very small, i.e. the attractive basin of noisy ICs is very small (Please refer to Appendix 2 for the details). Therefore, in practical applications, once all ICs are extracted no source can be extracted, which means ICA-R cannot produce any result which has correlation with reference. Hence if ICA-R cannot converge, it indicates that all ICs have been extracted. Specifically, when ICA-R runs up to a pre-set maximum step and does not converge, we consider that all ICs are recovered.

## Results

To demonstrate the effectiveness of the newly proposed ICA-R algorithm, both synthetic data and real-world data were used and the comparison of the results with the previous algorithms and other ICA methods were exhibited. In the following, we take four examples to illustrate the performance of our approach.

### Testing with self-supplied reference

**Figure 2:**
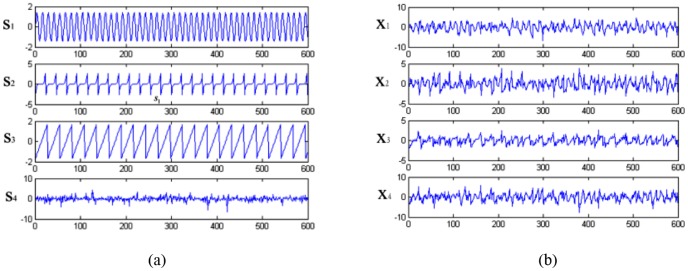
Results for synthetic data. (a) Four synthetic ICs (b) Observed signals.

Four synthetic sources were respectively depicted in Figure 2 (a), which could be considered independently to each other, where Sources 1 and 3 are with sub-Gaussian distribution, and the rests are with super-Gaussian distribution.

To linearly mix the four ICs, a special designed matrix was given as: 
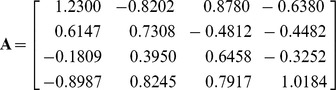
where there are two numbers in each line going to be multiplied by ICs of the same distribution, and only the absolute values of the diagonal elements are bigger than 0.5 when normalizing each line to unit length. For instance, the normalized second line was [0.5302, 0.6303, -0.4150, -0.3866] in which only the absolute value of 0.6303 was bigger than 0.5 to mix the super-Gaussian source (0.5302 would be used to multiply a sub-Gaussian source). In Figure 2 (b), the mixtures were shown as observed signals. Theoretically, if 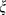 was set to −0.5, the desired IC could be extracted by ICA-R with reference, 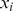 (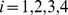). We tested the previous fast ICA-R algorithm on this problem instead of original ICA-R algorithm since they are inherently similar whereas the fast algorithm converges rapidly. In this experiment, we gave a random initial weight vector in each trial. Here, two fault learning were counted: misconvergence and negative Hessian matrix.

According to Table. 3, the probability that the previous fast ICA-R misconverged was higher than 10%. Although previous algorithm could produce accurate results even with the unguaranteed positive Hessian matrix, we still defined such learning process as a fault. Generally, the Newton-like optimization with a negative Hessian matrix should diverge, i.e. the weight vector grew fleetly (Lu's original ICA-R algorithm diverged in this case). However, for the every step normalization of weight vector, previous fast ICA-R would not diverge (still with probability of misconvergence); nevertheless the trajectory of the learning exhibited a period of the “haphazard” behavior.

**Table 3:** The comparison of fault convergences (two defective learning behaviors) and mean time (s) consumption between previous and new ICA-R algorithms on synthetic data.

		IC 1	IC 2	IC 3	IC 4
Faults of previous fast ICA-R	Number of Misconvergence	189	210	112	122
	Number of negative Hessian matrix	613	523	707	447
	Intersection of both faults	43	90	26	18
Misconvergence of new ICA-R		0	0	0	0
Mean CPU time of previous ICA-R for accurate convergence		0.097	0.105	0.130	0.121
Mean CPU time of new ICA-R		0.113	0.111	0.150	0.127

EACH ALGORITHM WAS RUN FOR 1000 TIMES WITH LEARNING RATE EQUAL TO 0.1.

Compared with the drawbacks of the previous algorithm including both faults above, the complete accurate extractions by our proposed ICA-R algorithm was dazzling. In the Table. 3, the mean CPU time of previous ICA-R excluded cases which committed any of the two types of fault convergences. For the new ICA-R algorithm, since steps for anti-misconvergence and anti-negative Hessian matrix were implemented, the computational consumption increased but not too much. Therefore, the added mechanism in the new algorithm is efficient and works well.

### Recovering images from the mixtures

**Figure 3:**
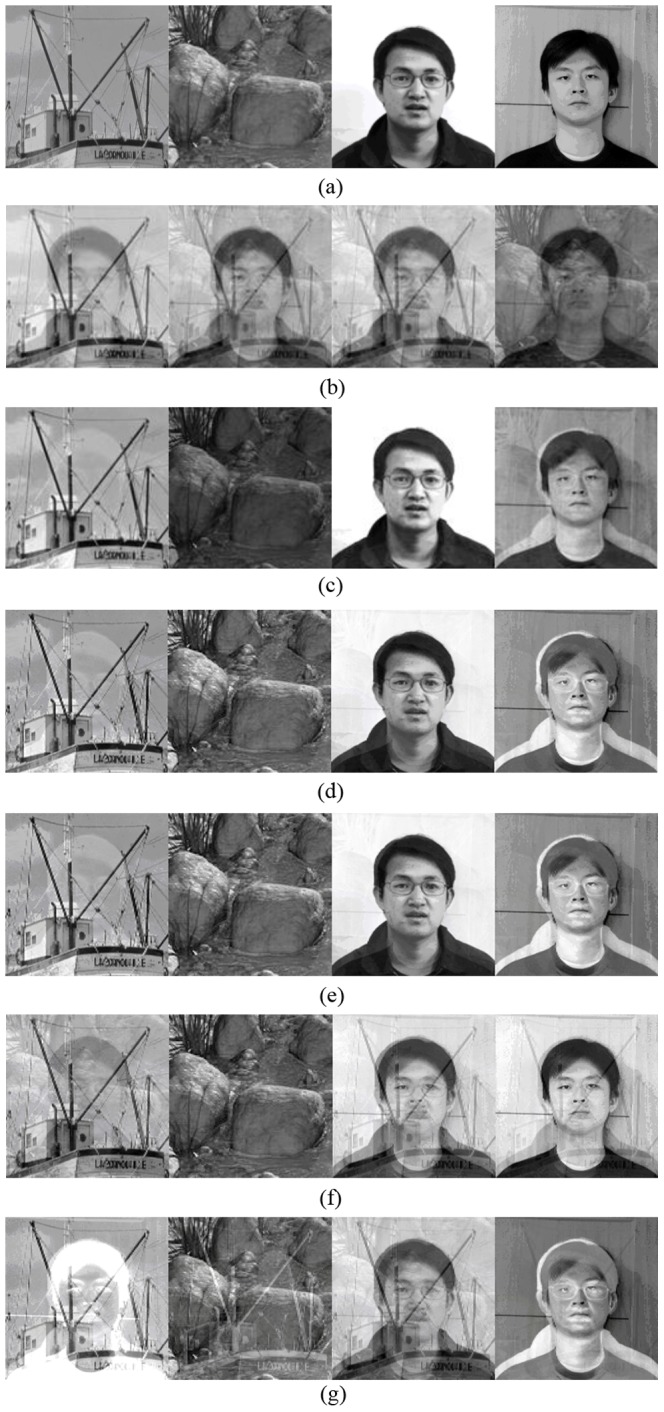
Results for image separation (The individual in this manuscript has given written informed consent (as outlined in PLOS consent form) to publish these case details). (a) Four original images; (b) Four mixture images; (c) Recovered images by new ICA-R; (d) Recovered images by FastICA; (e) Recovered images by EFICA; (f) Recovered images by JADE;(g) Recovered images by NGFICA.

There were two experiments conducted in this subsection, both of which were to recover all images from the mixtures. Since the images would have to be normalized, the signal-to-noise ratio (SNR) was used to measure the accuracy of each recovered image compared to its original image in dB 
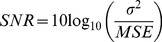
(10)where 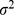 was the variance of the normalized images with the vector norm being 1. In the first trial, the four images, as illustrated in Figure 3 (a), containing two face images with size of 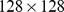 pixels, were mixed by a 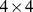 random mixing matrix to produce four mixture images, as illustrated in Figure 3 (b). The kurtosis of the original images were 2.5905, 1.6587, −1.3736 and −0.1626, respectively. The covariance matrix of the four original images was computed as: 
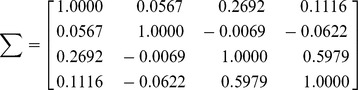
where there is a high correlation existing between the two face images. We used the new ICA-R algorithm to recover all the original images from their mixtures, where the first mixed image was used as the initial reference. And the results were shown in Figure 3 (c). To fairly compare it with the FastICA tools on SNR downloaded from [37], let the FastICA be fixed with symmetric orthogonalization approach to avoid the error cumulation. We also compared our method with other well-known ICA methods including EFICA (Efficient Variant of FastICA)[45], JADE[44], and NGFICA(Natural Gradient Flexible ICA) [24]. The EFICA is an improved vision of FastICA, which is believed to achieve better performance than FastICA. JADE and NGFICA are two widely-used classical ICA methods. The recovered images by different methods were shown in Figure 3(c)–(g) respectively. The comparisons of the SNRs of the outputs were given in Table 4. We can see that our method has the highest value of SNR for each recovered image and particularly the quality of the recovered face images by ICA-R is very good compared with others. EFICA produced the second best result in total SNR which indicates its superiority over its predecessor, FastICA. NGFICA may be incapable of recovering images from their mixtures, since it hardly recovered any clear IC.

**Table 4:** The comparison of the results of all the recovered images.

Algorithm		New ICA-R	FastICA	EFICA	JADE	NGFICA
SNR (dB)	Image 1	15.416	6.434	8.509	2.113	1.682
	Image 2	21.447	14.810	16.092	12.099	3.137
	Image 3	25.966	10.642	15.935	1.922	1.129
	Image 4	7.323	4.057	4.122	6.679	1.773
Total SNR		70.151	35.952	44.65	22.813	7.721

THE SNR RATE INDICATES THE SUPERIORITY OF THE NEW ICA-R ALGORITHM OVER THE CLASSICAL FASTICA GREATLY.

### Experiments on electrocardiogram (ECG) signals

The extraction of the fetal electrocardiogram (FECG) from maternal cutaneous electrode recordings has accepted ICA formulation [38]. To represent the superiority of the outputs by our algorithm, we used the famous ECG data set [39], and assumed nothing was known about the period of the FECG. Meanwhile, the results by other well-known ICA methods were shown for comparison.

**Figure 4:**
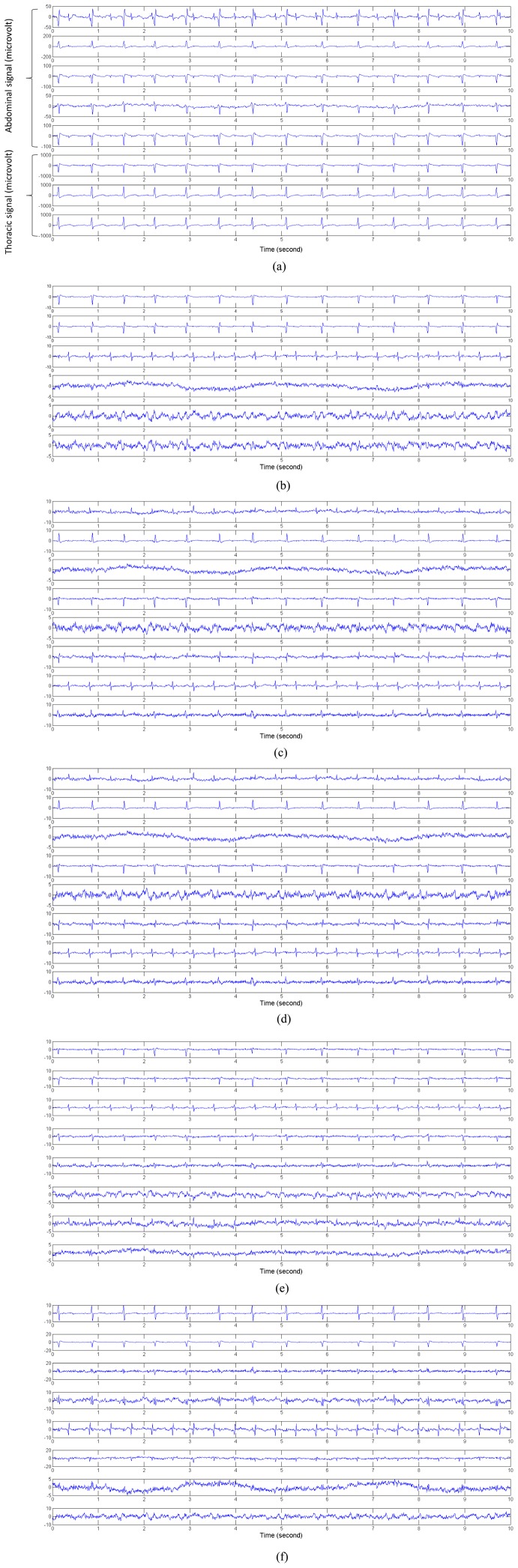
Results for ECG data. (a) The 8 channel ECG recordings obtained from a pregnant woman; (b) The results by our ICA-R algorithm; (c) The outputs by FastICA; (d) The outputs by EFICA; (e) The outputs by JADE; (f) The outputs by NGFICA.

First, our ICA-R algorithm was applied. From the observed ECG recordings shown in Figure 4 (a), the FECG can be obviously observed in the first channel, thus it can be used as the reference. Here, we set the closeness threshold parameter 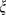 to −0.1 in our ICA-R algorithm. For the FECG was weaker than the maternal ECG (MECG), it was most likely that the ICs of the MECG were extracted first. By means of reference deflation, the FECG could be extracted for at most three runs of ICA-R algorithm. Two ICs of the MECG and one IC of the FECG were shown in the first three lines of Figure 4(b). And then, to explore the entire potential independent sources, we first summed all raw channels to produce a reference assumedly consisting of all potential ICs. Then, 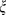 was reduced to −0.05 and the maximum running step was augmented to 10000. The first three sources in Figure 4 (b) could be easily extracted within 150 learning steps while there were around 300 iterations of learning required for the fourth source. It was very hard to extract next 2 sources within 1000 steps (sometimes 10000 steps of learning were even not enough). And by repeatedly running the ICA-R algorithm, it could be confirmed that only 6 possible sources could be extracted. The kurtosis values of 6 sources were 24.332, 20.409, 7.6149, −0.657, −0.391 and −0.2906, respectively. For the last two low absolute values of kurtosis, the corresponding extracted results were most likely to be noise. Further, we could deduce that there were not more than four ICs existing in the observed signals (under the assumption that ICs are of nongaussian distribution). Actually, the first two extracted sources were the MECGs while the third one was the FECG, and the fourth one perhaps was produced by breath or some other causes. The correlation coefficient between two MECG sources was computed, which was 0.305, and the correlation coefficients between FMCG sources and two MECG sources were −0.0066 and 0.0067, respectively. From these results it can be seen that the existing correlation between two MECG sources as well as the independence between MECG sources and FMCG sources were reasonable.

We can see that the results produced by FastICA and EFICA (shown in Figure 4(c) and (d)) are very similar. JADE (shown in Figure 4 (e)) did not extract a source, which may be caused by breath, as others. NFGICA had good performance (shown in Figure 4 (f)) in this case that the first and second ICs are clear. Compared with the results of our ICA-R, it can intuitively reveal that the results for FastICA and EFICA were of lower quality, which probably was caused by the sensor noises. Except ICA-R, classical algorithms have to produce the same number of the outputs as the number of the inputs. However, it was very hard to give a rational physiological explanation about what the mass-produced ICs stand for. It could be concluded from this experiment that in practical applications besides extracting ICs with high quality, our algorithm was also capable of helping to reveal the number of underlying sources when the observed signals are more than ICs but others were not. Moreover, unlike the results by our algorithm which maintains the inherent correlation between two MECG sources, the results of FastICA were uncorrelated to each other completely. Thus, at this point, our algorithm has superiority over FastICA on preserving essential property of the original underlying sources.

**Remark 2:** In practical applications, how is the learning rate of 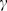 selected? This is an important problem. Although it is not difficult to choose a proper learning rate 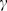 (typically 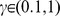), what still have to be noted is that too small value of 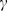 can make the algorithm learn slowly while too large value of 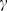 may cause the algorithm to ignore some ICs with small negentropy values. A strategy is to set up a big learning rate at first, and if the algorithm does not converge in a given number of iterations, then the rate is reduced in half.

## Conclusions

The cICA framework of incorporating the extra requirement as constraints into ICA contrast function is an attractive method. In this paper, a new ICA-R algorithm was proposed under the cICA framework and applied to solve some independent components extraction problems. The ICA-R algorithm is an optimization algorithm in which the minimum of the objective function is searched by Newton-like learning. It has been shown that the previous ICA-R algorithms cannot guarantee convergence to the desired independent components but may produce fake ICs. Our proposed new algorithm fixes this problem by adding a few extra steps to prevent the misconvergence. This ensures that only the true ICs are extracted.

Another contribution of this paper is to facilitate and extend the applicability of the ICA-R algorithm. A reference deflation technique was introduced to help choose the critical parameter, the closeness threshold. A simple approach was proposed to provide the ICA-R algorithm with a direct reference from the observed signals instead of process of using an ad hoc reference construction. The above two techniques could broaden the applicability of the ICA-R algorithm. The proposed one-unit ICA-R algorithm is also extended to recover all of ICs from their mixture. Although the original ICA-R algorithm was designed to extract only one IC of interest, the successful application of recovering all of ICs using the new ICA-R algorithm indicates that our approach improves significantly upon the previous work. Compared to FastICA, we have shown that our approach is advantageous in four ways: firstly, no error accumulates when iteratively recovering the ICs; secondly, no compulsive decorrelation is required between the outputs which could help to maintain the original underlying sources; thirdly, our new algorithm can aid determination of the exact number of the true ICs in the case where the number of the observed signals is more than underlying components; fourthly, when sensor noise is present, our new algorithm can produce the results that are less affected than FastICA. All the advantages mentioned above have been validated in the previous experimental section, which also opens and develops the potential application fields for the ICA-R algorithms.

In conclusion, from all the above encouraging results and analyses, the proposed ICA-R algorithms can not only extract the desired ICs reliably and efficiently but also is appealing for enabling wide use of ICA-R.

## Appendix 1

### Introduction to ICA with reference

According to [3], ICA-R is a constrained optimization problem as follows: 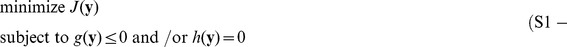
(S1<?ENTCHAR hyphen?>1)where 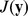 denotes the ICA contrast function approximating negentropy, which equals 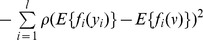 with 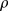 being a positive constant and 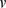 a Gaussian variable having zero mean and unit variance, and 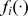 is a nonquadratic function; 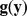 is the inequality constraint term, 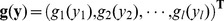 with 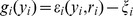, where 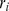 is the related reference signal, 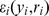 is the closeness measurement between the output and the reference signal, and 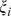 is the threshold parameter; 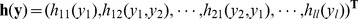 is the equality constraints term, where 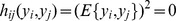,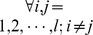 and 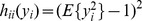, 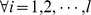. There are generally two version functions, 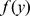, chosen as the negentropy approximation function. These are the super-Gaussian and sub-Gaussian, respectively: 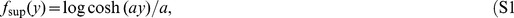
(S1<?ENTCHAR hyphen?>2)

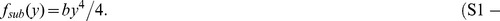
(S1<?ENTCHAR hyphen?>3)


The corresponding augmented Lagrangian function is: 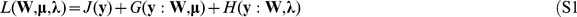
(S1<?ENTCHAR hyphen?>4)where 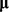 and 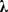 are Lagrangian multipliers for inequality and equality constraints, respectively; 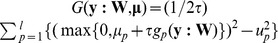 and 
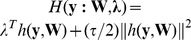
 with 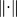 denoting the Euclidean norm and 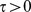 being the penalty parameter.

## Appendix 2

### The derivation of the attractive basin of an IC

Given a linear mixture 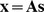, where ICs are assumed to be vectors with zero mean and unit variance, and assume 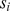 to be the target IC with 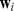 (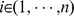) being the corresponding demixing vector. Then we have: 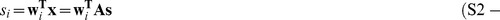
(S2<?ENTCHAR hyphen?>1)



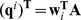
 is a transformed vector, where 
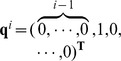
 and 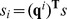. To find the basin of attraction of 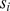 in the feasible space of the equality constraint, where 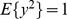, we could alternatively measure the size of area in which the gradient of 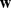, 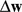, is pointing to 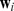. When there is no inequality constraint, the gradient 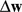 can be written as: 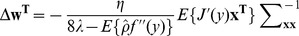
(S2<?ENTCHAR hyphen?>2)


Given a vector, 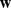, in the equality constraint space, we note 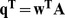, so: 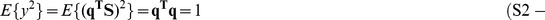
(S2<?ENTCHAR hyphen?>3)where 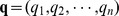. Based on Eqn.(S1-2), the gradient of 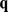 is: 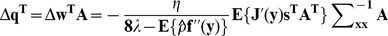
(S2<?ENTCHAR hyphen?>4)


Since 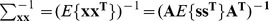, based on the independence property of 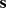, there is a 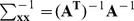. Therefore, we have: 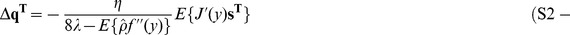
(S2<?ENTCHAR hyphen?>5)


For the simplicity and without loss of generality, we let 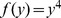 and negentropy approximation function 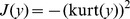 where 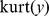 is the kurtosis of 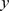 defined by 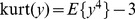. Since only the direction, i.e. 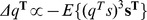 is concerned then by the independence property we have: 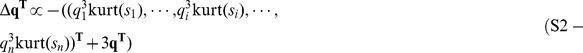
(S2<?ENTCHAR hyphen?>6)


Since the second term in the right side of Eqn.(S1-6), 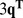, only prolongs the length of 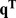, so 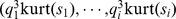 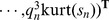 determines the gradient. This is easily derived that when 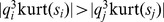 (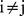, 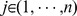), 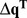 will point to 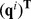 and 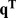 will converge towards 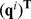.

Then, the condition is: 
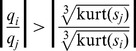
(S2<?ENTCHAR hyphen?>7)


So the region in the feasible space of the equality constraint which satisfies Eqn. (S2-7) is the attractive basin of 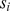. It can be concluded that the IC's attractive region is related to its relative value of the corresponding negentropy. If an IC has a larger negentropy than the others, it will have larger attractive basin in the feasible space of equality constraint and vice versa.

## Appendix 3

### The derivation of the center of inequality constraint

If iteration of ICA-R is outside of the feasible region of inequality constraint then the algorithm will draw the learning into the feasible region, towards the center of inequality constraint, by increasing the Lagrangian multiplier 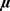. Generally, the closeness measures can be selected as 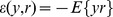, 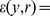 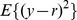, 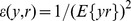, and so on, which can be expressed as 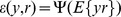 where 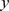 and 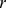 have zero mean and unit variance. The center of the feasible region of the inequality constraint is 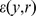, which can be found by solving the following optimization problem: 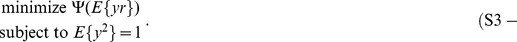
(S3<?ENTCHAR hyphen?>1)


The corresponding Lagrangian function is given by: 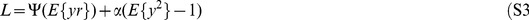
(S3<?ENTCHAR hyphen?>2)


Then, we let: 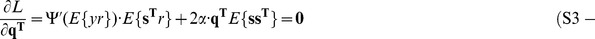
(S3<?ENTCHAR hyphen?>3)where 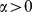. And since only the direction is of interest: 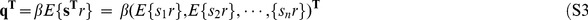
(S3<?ENTCHAR hyphen?>4)where 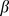 is some constant that makes 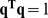 satisfied. Thus the center is determined by the correlation between the reference signal and the ICs.
